# On Contagion

**Published:** 1825-05

**Authors:** Charles Larkin


THE LONDON
### Medical and Physical Journal.

No. 5 OF VOL. LIII.] MAY, 1825. [No. 315.

For many fortunate discoveries in medicine, and for the detection of numerous errors, the world is indebted to the rapid circulation of Monthly Journals; and there never existed any work, to which the Faculty, in Europe and America, were under deeper obligations, than to the Medical and Physical Journal of London, now forming a long, but an invaluable, series.—RUSH.

### ORIGINAL COMMUNICATIONS,
### SELECT OBSERVATIONS, &c.

### ART. I.—*On Contagion.* By CHARLES LARKIN, Esq.

[Continued from page 284.]

IN the former part of this paper, I was engaged in the explication of some of the various senses which have been attached to the words contagion and infection; an explication which it was necessary to enter into, for these verbal ambiguities have tended to throw a haze about this question, which does not naturally belong to it;—and in a cursorary examination (for the limits I have prescribed to myself will not permit me to enter into a more extended review,) of the *facts* which have been adduced to prove the contagious and importable nature of plague. In this part, I shall endeavour to collect a few facts and observations, which are totally inconsistent with, and entirely inexplicable upon, this hypothesis; forming an index that points to some cause and origin of the disease, other than contagion. I shall arrange what I have to say under five heads.

1. My first objection, then, to the doctrine of contagion, is grounded upon a maxim which has received the universal assent of philosophers,—that causes ought not to be unnecessarily multiplied. It must be admitted that plague, and every other disease, had, in the first instance, another origin than contagion. Whatever, therefore, that cause was that first produced plague, or any other disease, that cause, I do assert, still continues to keep the disease alive, in spite of quarantine and precautionary regulations.

2. My second objection is, that it has no foundation in observation and experience. This I have already endeavoured to show.

3. My third objection is, that observation and experience are directly against it. In support of this objection, I invite attention to the following facts.

In many of the places which the plague has visited, it has confined its attacks to the natives, and left foreigners untouched. We learn from Sir A. B. Faulkner that this was the case at Malta. He tells us that the soldiers did not obey the orders prohibiting them from any intercourse with the inhabitants; yet in one regiment only one man took the plague, *and he infected none of the soldiers in the same company;* and among all the troops not more than twenty died. This is a curious, but not a singular fact. While the plague was at Bath, no Italians, Germans, or Frenchmen, became the subjects of the disease. At Hafni, in Denmark, during a pestilence, all strangers escaped, *though they lived promiscuously in the infected habitations.* The sweating sickness of 1485 attacked only Englishmen, who did not escape by travelling into France and Flanders. A similar fact occurred in the great pestilential visitation of 1348. The disease was first discovered in the empire of Cathai, in the north-eastern part of Asia, whence its course was traced through the different provinces of that continent to the Delta and banks of the Nile: a south wind transported it into Greece and the Grecian Islands; thence it swept the coast of the Mediterranean, depopulated Italy, and crossed the barrier of the Alps into France. A succession of earthquakes, which shook Europe from Calabria to the north of Poland, ushered in this fatal year; and, though England escaped this calamity, it was deluged, from June to December, with almost incessant torrents of rain. Do not these circumstances indicate something amiss in the elements above, around, and beneath us, with which we are made to sympathise? In the first week of August, the plague made its appearance at Dorchester,—in November it reached London,—and thence gradually proceeded to the north of the island. Of its victims, many expired in the course of six hours, and few lingered more than two or three days. Men were not the only victims of this exterminating malady; destruction fell upon the brute creation, and the carcases of sheep, horses, and oxen, lay scattered in the fields. Birds of prey left them untouched, and their putrefaction aggravated the malignity of the disorder. The labours of husbandry were neglected. No courts of justice were opened. The Parliament was repeatedly prorogued by proclamation; and men, intent only on their own safety, fled from the care of the infected, and slighted every call of honour, duty, and humanity. The mortality occasioned by this wide-wasting pestilence, is by some historians estimated at a third, by others at one-half, of the human race.

I particularise these facts, because they show, in the most clear and convincing manner, that the historians of this period had no idea that plague could be transported in goods or merchandise of any description; but that they considered the winds of heaven to be the bearers of infection to the several countries of the globe. The same appears to have been the idea of Procopius, who represents the plague which he describes, and which first appeared in the neighbourhood of Pelusium, between the Serbonian bog and eastern channel of the Nile, as travelling by a double path; spreading from this point to the east, over Syria, Persia, and the Indies, and penetrating to the west along the coast of Africa, and over the continent of Europe.

To return from this digression. This malady, though it assailed the English in Ireland, spared the natives. Scotland also escaped for several months; and this circumstance afforded them a subject of triumph over their enemies, and introduced among them a popular oath, "By the foul dethe of the English." It reached, however, these presumptuous and insolent men at last, and invaded their camp in the forest of Selkirk, at the very time when they had assembled an army to invade the neighbouring counties, and to extend the ravages of pestilence by the flames and sword of war. Five thousand died before they disbanded their forces; and the disease penetrated at length into the most distant recesses of the country. These are of themselves strong facts against the doctrine of contagion; but I have much stronger in reserve.

Sir R. Wilson, a perfectly unprejudiced witness, tells us, in the evidence which he gave before the Committee of the House of Commons, that the army which invaded Egypt was divided into two corps; one was stationed at Alexandria, and the other moved on against Cairo. "That part of the army, Turkish and British, which moved against Cairo, passed through the country where numerous villages were infected with the plague; and, during the march, the soldiers had constant communication with the inhabitants of those infected villages. At Menoof, where the plague raged with the greatest violence, a bakery was necessarily established for the use of the army; but none of the persons who attended that bakery were infected with the plague. At Rahmanich there was a plague-hospital; several men were lying infected with the plague, and many were brought out already dead; others were dying in the environs of the town, of the same disorder: the Turks stripped the bodies of all indiscriminately of their clothing, and there was no restraint whatsoever in the communication with the inhabitants, who had also free access to the camps; *yet no plague was communicated to the troops.* It was also affirmed to us by the French officers, that, although the plague had raged in Cairo that year with very great violence, and carried off some of the French army, yet, notwithstanding a constant communication was held between the garrison stationed in the citadel and the inhabitants of the town, the soldiers of the citadel were not affected, in any one instance, with the disorder. I would also wish to state, that, as we moved through the country, the inhabitants pointed out to us particular villages that were infected with the plague, and which plague did not extend out of those particular villages to any of the contiguous villages, *although there was no precaution whatever used as to the communication with the inhabitants of the infected villages !*" He also states, that the Indian army, passing through Upper Egypt, had traversed a country in which 60,000 inhabitants were said to have perished,—whole villages having been destroyed; yet the troops of that army brought no infection with them, "*nor were any precautions adopted to prevent contagion on their junction with the British European army.*" There are several other very strong and interesting facts in the evidence of this gentleman, which want of room compels us to withhold.

Dr. Russel, whose laborious work upon the Plague is remarkable for its general candour and fairness, observes, that, in winter time, when infected persons have come to places about Aleppo, some of whom have died in families where they lodged, the distemper was not by such means propagated. Evagrius also, in his Ecclesiastical History, informs us, that many of those who left infected places were seized with plague in the towns to which they had retired, *without communicating it to any of the other inhabitants.* The following passage from Dr. Russel is deserving of serious attention:—"Experience in Turkey, where generally no precautions are taken in time of pestilence, clearly evinces that, in a certain state of the air, a communication with infected places may subsist without any material consequence. The return of the plague at Aleppo happens at irregular periods; the intervals are of considerable, but unequal length; *and in those the commerce with Egypt, Constantinople, and Smyrna, remains uninterrupted. In the intervals between* 1744 *and* 1760, *and from* 1762 *to* 1780, *the plague raged several times in the places now mentioned, without affecting Aleppo; and even in two or three years subsequent to* 1762, *though it was at Marash, as well as other places not far distant, with which Aleppo has continual intercourse, no instances were discovered of communicated infection. I consider it, therefore, as an established fact in the Levant, that commerce and intercourse with infected towns is sometimes attended with no bad consequence.*" Strange this, indeed, if, as Sir A. B. Faulkner, Dr. Granville, and the Committee of the House of Commons, tell us, the plague be *communicable by contact alone!*

From this passage we learn that, in general, no precautions are taken by the Turks in times of pestilence. This is a circumstance which I cannot help remarking upon. If plague be contagious, it is something more than extraordinary that this people should be so blind to what is passing beneath their eyes, that men, who are more familiar with the plague than we are with the commonest disorder that prevails amongst us, should be so infatuated as, notwithstanding the experience of almost every day, not only to disbelieve, but positively deny, the existence of contagion; the existence of which, the contagionists assure us, is as glaring and notorious as the mid-day sun. I can scarcely believe that a set of abstract religious notions about fate and predestination could blind a whole people to a notorious fact,—take away from them all concern about their own safety, —fill them with such an incredible degree of apathy, and so utterly deprive them of reason, that they should stand and stupidly gaze upon evils, which daily observation tells them are not only not remediless, but aggravated by their own want of caution, without an effort to avoid or to avert them. This is a state of horrible fascination, in which I cannot believe it possible that a whole people could be held for so many ages. If they have been, I can only account for it by supposing that God has first driven mad those whom he has wished to destroy. The belief in fate and predestination is not peculiar nor confined to the Turks, but has at all times been prevalent, and forms a very material part of some systems of philosophy in vogue at present, and a fundamental article in the creeds of many Christian sects. Yet among these it produces no such apathy, and no such scepticism as to the real existence of what they see. They believe, and are yet not insensible to danger. Why, therefore, a faith of this kind should produce effects in Turkey which it does not give rise to in other parts of the world, I cannot conceive. It is not from his belief in fate and predestination, that the apparent supineness of the Turk arises, but from a conviction, founded upon long experience, and the observation of centuries, that plague is not contagious, and that mounds, and fortifications, and armed troops, are of no avail against so subtle and invisible an enemy. And I am the more confirmed in this belief, because we learn from Procopius that the inhabitants of Constantinople in his day were equally assured that there was no danger in the closest approximation to the sick. The Christian of that day, like the Turk of the present, approached the bed of the plague-stricken man without fear, and boldly performed all the duties of humanity. Physicians then did not feel the sick man's pulse through a tobacco-leaf, or leaf of any other kind; nor did they ascend an elevation of ten or fifteen feet above the patients for whom they prescribed, in order to escape the pestiferous effluvia which they supposed were issuing from every pore of the sick man's skin; a notion which I believe to be as purely fabulous as the idea of dragons, whose breath was flame, or of basilisks, whose glance was mortal.

But to return.—To the authorities quoted above, I have now to add that of Dr. Hodges, another eminent contagionist. In his account of the Plague of London, he states, that when, upon the decline of the disease, the thousands, who had deserted their homes and their duty, returned to their habitations, such a degree of confidence and temerity succeeded to the terror that had agitated the breasts of these wretched beings, that they actually slept in the same beds in which some of their friends had died of the malady, before those beds had become cold, or were cleansed from the stench of the disease; *yet they remained free from infection.*

Sir R. Wilson, in his evidence before the Committee of the House of Commons, has noticed the exemption of particular villages from the plague, whilst it was raging in contiguous villages. Dr. Russel, in a passage which I have quoted, also instances the freedom of Aleppo from pestilence, while it was laying waste its vicinity. During the last plague that desolated England, Oxford was exempt. To these examples of particular villages and particular towns, I shall now append the instance of a large kingdom. Plague, if not always, has at least generally respected the territory of Persia, though it is completely surrounded, and has continual intercourse, with countries in which this dreadful enemy of the human race is running riot with unrestrained energies and unmuzzled fury.

Either, then, these facts, whose verity is admitted by the contagionist, and supported on authority too high to be discredited, are not true, or the doctrine of contagion is false. But as brave men never lose heart, even in the last extremity, so neither is an ingenious man, however hard pressed, ever at a loss for some substitute or other for an argument. The contagionist has always an assumption to cover his retreat with, and can work wonders with the doctrine of insusceptibility. "If," says Dr. Russel, "of one hundred persons exposed to the infection of plague by a near approach to the sick, ninety should fall ill, *shall human inability to assign* SATISFACTORY REASONS for the preservation of the other ten, be converted into a positive argument against the disease having been caught by contagion?" Is this a fair statement of the argument of the anti-contagionist? Is it not rather a glaring perversion of the facts? Had Dr. Russel shown this to be the case at all times, in all places, and under all circumstances, then would I grant that it was correctly put, and I would retire from the contest, silent and defeated. But I deny his ability to do so, until he can, like another Omar, burn all the records of history, extinguish in man the faculty of memory, and then be at liberty to forge facts adapted to the end which he has in view. This insusceptibility I have shown to exist, not only in ten men out of every hundred, but in whole companies and regiments of men,—not only in whole companies and regiments of men, but in a great army,—not only in a great army, but in entire villages and towns,—not only in entire villages and towns, but in an empire, wide-extended, and crowded with inhabitants! This surely is to give a very different face to the argument.

There are some other facts belonging to this part of my subject, which I hold to be inexplicable on this hypothesis. In its translation from one place to another, plague is very irregular and capricious. Thuanus mentions an instance of a plague, which was one year at Trent and Verona, the next at Venice and Padua, leaving Vicenza, an intermediate place, uninfected. During the plague at Marseilles, a similar instance occurred, when the plague was carried at once out of Provence, several leagues into the Gevaudan. Mr. Legh, in his travels through Egypt and Nubia, informs us that he was obliged to abandon his original design of travelling by Smyrna to the capital of the Eastern Empire, on account of the plague, which was rife at Constantinople in 1812, and throughout Asia Minor, and proceed towards Egypt; for, though the communication between Constantinople and Alexandria had been uninterrupted, *the latter remained perfectly free from contagion*: and so strange and inexplicable is the mode in which this dreadful disorder spreads from one country to another, that a Greek, who acted as British consul at Scio, observed to him, *he had no fear of the infection being communicated from Smyrna, where numbers were daily dying, and from which persons were daily arriving at the island, though within a few hours' sail; but, he added, should the plague declare itself at Alexandria, distant several hundred miles, we shall inevitably have it at Scio.* The solution of these difficulties I leave to others; it is sufficient for me that they point to the existence of some other cause of plague than any which is dreamt of in the philosophy of the contagionist.

4. My fourth objection to the doctrine of contagion, is one which its advocates have felt, and acknowledged the force of. It is this: If plague be communicable solely by contact, as long as there are human susceptible creatures to work upon, it never could cease, especially when such numerous fountains of the evil were opened upon us, as when the disease prevails to a great extent in the metropolis of a large and populous empire. Dr. Mead and Dr. Russel not only admit the validity of this objection, but state it in very pointed terms. "A corrupted state of the air," says the former of these gentlemen, "is without doubt necessary to give these contagious atoms their full force; *for otherwise it were not easy to conceive how the plague, when once it had seized any place, should ever cease but with the destruction of all the inhabitants;* which is readily accounted for by supposing an emendation of the qualities of the air, and the restoring of it to a healthful state, capable of dissipating and suppressing the malignity." The objection is, however, stated with much greater emphasis by Dr. Russell: "But a greater difficulty than that of all persons not being equally susceptible of infection, arises from the cessation of the plague, *at a period when the supposed contagious effluvia, preserved in apparel, furniture, and other fomites, at the end of a pestilential season, must be allowed, not only to exist in a much greater quantity than can be supposed to be at once accidentally imported by commerce, but in a state of universal dispersion over the city.* The fact, however unaccountable, is unquestionably certain: the distemper seems to be extinguished by some cause or causes, equally unknown as those which concurred to render it more or less epidemical in its advance and at its height. In Europe, something may be ascribed to the means employed for the cleansing of houses and goods, supposed liable to retain the latent seeds of infection; *but at Aleppo, where the distemper is left to take its natural course, and few or no means of purification are employed, it pursues nearly the same progress in different years: it declines and revives in certain seasons, and at length, without the interference of human aid, ceases entirely.*" I have preferred stating this objection in the words of the contagionists, because I feel sensible that, coming from such authorities, it will have much more weight and force, than if I stated it as directly emanating from myself. I shall not further enlarge upon it, but leave it to produce its own infallible effect upon every unprejudiced and candid mind.

5. Such are the facts which I place in direct opposition to the doctrine of contagion, and which I could easily multiply, had I a larger space to expatiate in, and were I not afraid of fatiguing the patience of my reader. The witnesses whom I have brought forward, with the exception of Sir R. Wilson and Mr. Legh, are the contagionists themselves: they stand vouchers for the verity of my facts. I now then proceed to my fifth objection. As the contagionist attempts indirectly to prove the truth of his creed by the efficiency of the precautionary regulations, which, in conformity with his doctrine, he urges the adoption of, I shall attempt to prove its falsity by demonstrating, very shortly, the utter inefficiency of that system of quarantine, which is his boast, his pride, and his glory. And here, again, the facts which I shall adduce shall be the admissions of the contagionist: the language I shall use shall be his. "Non meus hic sermo, sed quæ precepit Ofellus, rusticus abnormis sapiens." Allow me, then, again to direct my reader's attention to Malta, and to the facts stated by Dr. Faulkner himself, with regard to the decline of the plague in that island.

The system of police began to be organised on the 3d of July. On the 16th day of the same month, the disorder was at its height; and from this period it began visibly to decline. The police was not fully organised until the 2d of August; nor were the inhabitants, until that period, shut up, or prohibited from mutual intercourse or contact. On the 16th of July, sixty-seven died; on the 2d of August, fifty; on the 13th of August, thirty-one; and it was not until the 23d of October that the disease finally disappeared. Perpend this matter a little. From the 16th of July to the 2d of August,—that is, rather more than a fortnight previous to the complete organisation of the police system, and before the inhabitants were shut up and interdicted mutual intercourse, the decrease in the number of deaths is from sixty-seven to fifty:—*that is, before these regulations could, by possibility, have the slightest influence in checking the course of the disease, there is a decrease of seventeen in the number of deaths.* The police system is established on the 2d of August; the inhabitants are confined to their houses, and all intercourse prevented. Is the decrease in the number of deaths more apparent? No. In spite of these regulations, the disease holds a steady and gradual course, and a little less than a fortnight after they had been enforced, the decrease is as follows:—2d of August, fifty; 13th ditto, thirty-one; a decrease of nineteen. The period when the disease had actually and visibly begun to decline, is cunningly selected for the establishment of a system of police, and for shutting up the inhabitants in their houses, for no other purpose but that of perpetuating delusion, and of robbing nature of the glory of that which she had really effectuated by her own unaided exertions. Notwithstanding this police system, nearly three months elapse before the work of destruction ceases. The inference from these facts is so plain, that I will not insult the understanding of my reader by formally stating it. I may, however, just observe, in passing, that the very occurrence of plague in Malta strikingly demonstrates the insufficiency of this boasted and vaunted system. From Malta let us turn our eyes to London.

In the last plague, the same fact of the utter feebleness of all regulations, is equally well attested. We have the concurrent testimony of every writer on the subject, that, in spite of every regulation, though they were early and strictly enforced,—enforced with rigour and cruelty,—in defiance, in pure scorn, of all the efforts of helpless man,—the disease posted on with unremitting speed, and was as resistless as the element which fed upon their habitations the year after the plague had devoured the inhabitants. It began in December, appeared and disappeared by fits. About June it fully and openly declared itself; and from this time, though every precaution which the mind of man could devise was instantly and boldly put in execution, it daily and hourly increased; and, to quote the simple but powerful language of the journalist who has transmitted to posterity an account of this dreadful visitation, "to such a violence did the plague come at last, that the people sat still looking at one another, and seemed quite abandoned to despair. Whole streets seemed to be desolated, and not to be shut up only, but to be quite emptied of inhabitants; doors were left open, windows stood shattering with the wind, in empty houses, for want of people to shut them : in a word, people began to give themselves up to their fears, and to think all regulations and all precautions were vain, and that nothing else was to be hoped for but universal desolation." It was then, in the midst of this state of universal despondency, when every person that was seized with the distemper died, and when in one night 3,000 persons were wrenched from all they loved, and when 12,000 perished in one week,—when the people began to despise all caution, and the cruel rigour of the temporary laws was relaxed, and there was no hope for safety but in Divine interposition,—that, in the words of the journalist, "it pleased God to stay his hand, and slacken the fury of the contagion, *and in such a manner as demonstrated it to be his own particular hand.*" No expression could more strongly evince, than this, the conviction which the journalist had, that all the precautions which had been used were useless, and all the barriers which had been erected to check its progress were of no more avail than it would have been to have attempted to stop the ocean with a bulrush. In the last week of September, the plague, being come to a crisis, began to abate its fury. The distemper became milder, and, though the numbers of infected continued the same, the mortality was less. In this week there was a decrease of 2,000 in the Bills of Mortality. Things changed greatly. *Out of* 60,000 *infected*, 40,000 *recovered; whereas, before, out of that number* 50,000 *would have died.* These facts are so eloquent, that, to comment upon them, or attempt to add to their force by any observations of my own, would be equally absurd and ridiculous.

In confirmation, however, of the truth of what I have stated, I may be permitted to quote the following passage from Dr. Hodges. "As soon as the magistrates saw how the contagion daily increased, and had extended itself to several parishes, an order was immediately issued to shut up all infected houses; but, *whether this method proved of service or not, is to this day doubtful and much disputed.* The houses of infected persons were marked with a large red cross, with this inscription, 'Lord, have mercy on us!'—a guard attended continually, both to hand to the sick food and medicine, and to restrain them from coming abroad till forty days after their recovery. *But, though the Lord Mayor, and all inferior officers, effectually put these orders into execution, it was to no purpose, for the plague more and more increased.*"

We learn from Dr. Mead, that plague constantly ceases at Smyrna about the 24th of June, without the aid of any regulations. Dr. Russel also bears testimony to the same fact with respect to Aleppo. The same phenomena were observed at Marseilles. "We have seen," says Dr. Russel, "that the plague at Marseilles began to decline long before it was in the power of the police to attempt the general expurgation of the city, which took place in the sequel; *and that the measures taken for that purpose, however rigorously pursued, did not succeed in extinguishing the distemper, which continued lingering several months after.* It declined, he says, with a rapidity not ascribable to the exertions of the most vigorous police!"

If any thing can, these facts surely do prove the vanity and futility of all the means by which the contagionist attempts to arrest the progress of plague. As well might he attempt to hide the sun behind a blanket, or stop the diurnal revolution of the earth! A sarcastic philosopher among the ancients has observed, that the chief amusement of the gods consists in beholding the comedy of human life, and laughing at the grave solemnity with which man attempts impossibilities; and that their chief employment consists in frustrating all his mighty schemes, and rendering vain and nugatory all his laborious efforts, dooming him to realise the melancholy fables of Sisyphus and the unhappy daughters of Danaus, by enduing him with a disposition never to despair, though always defeated, and by perpetually renovating hopes, which they as perpetually disappoint. If this be true, the contagionists, with their system of quarantine, have certainly furnished forth one of the richest scenes of the drama. We have seen how plague has baffled all their attempts to arrest it; we shall now see that their attempts to barricado it out are equally inefficacious.

Apolini states, that he has known numerous instances of people being infected, who were shut up and completely segregated from all intercourse with the contaminated. It was also noticed, during the plague of Marseilles in 1720, that those who shut themselves up most securely, and were most careful in taking nothing without the most exact precaution, were attacked with the disease, which creeps in no one knows how. Dr. Russel also admits that he met with several instances of the like kind at Aleppo, in the houses of the Christian and Jewish natives. In the "Relation Historique" of the Plague of Marseilles, the fact is stated more explicitly. We are told that, in the height of the pestilence, the infection penetrated into places which till then had remained inaccessible, and that monasteries and houses, shut up in the most exact manner, were no longer places of security. Sir R. Wilson has also told us, that the division of the army which was stationed at Alexandria had a detachment at Aboukir, *and that, notwithstanding every precaution was taken to prevent the introduction of the plague into that part of the army which blockaded Alexandria, and was stationed at Aboukir, and, from particular local circumstances, all communication with the country was successfully intercepted, except under authorised regulations,—notwithstanding these precautions,* PLAGUE BROKE OUT THREE DISTINCT TIMES. Had I to accumulate facts till doomsday, I could not make this subject clearer than it is made by these incontrovertible testimonies. In order to give them, however, full force and efficacy, I shall conclude them with the following remarkable sentence from Dr. Russel, who observes, that quarantine and other regulations have often proved ineffectual in arresting the progress of plague; *that it has frequently occurred insidiously when they have been rigidly enforced, and in a more extraordinary manner has ceased when they have been entirely relaxed.*

It is impossible, in a paper of this kind, without far overstepping the boundaries within which I wish to confine myself, to enter into a review of the Sanitary Laws, and the whole system of quarantine. I may be allowed to say, however, that never did a system exist better adapted for all the purposes of tyranny, and worse calculated for the preservation and security of public health. Instead of warding off pestilence, were it capable of importation, it were impossible for the very spirit of evil to have devised a system better fitted to introduce it wheresoever that system prevails. It treacherously opens those very gates which it is appointed to guard, to the enemy, from whom it pretends to defend us; and this, had I room, I would undertake to prove to the full satisfaction of every man.

To mention a few facts corroborative of what I assert. Bills of health give us no information of the real condition of merchandise with respect to contagion. If two ships be laden with clean cargoes in a period of health, and one should happen to sail a day or two before the other, should a single case of plague occur in the port or city in the mean time, the other vessel, although she had no communication with the shore, is obliged to sail with a foul bill. Here, though the supposed danger is equal in both cases, one is immediately released, and the other condemned to undergo the whole of the expurgatory process, and the rigour of the full period of quarantine. Should two ships be laden with foul cargoes during pestilence, and one sail with a foul bill of health thirty days after the plague has ceased, and the other waits ten days more to be entitled to a clean bill, the first (though both must, of necessity, be equally infected,) is obliged to perform quarantine; while the other brings her contaminated cargo at once into the market. It is unnecessary to multiply instances. "The risk," says Dr. Russel, "arising from the chance of ships being dispatched with clean patents, is assuredly not ideal: as things stand at present, bills of health are not entitled to that degree of credit they ought to have. A clean patent at certain times brings, in reality, less security to the kingdom than one touched, or expressive of suspicion. The one puts the council of health on its guard, the other fills it with imaginary security; and, as long as clean patents only are admitted into England, it may justly be apprehended that, under the mask of that title, such as ought in reality to have been reckoned touched, if not foul, will sometimes find their way, in spite of any thing in the power of consuls abroad, under the present constitution, to hinder." Yet, notwithstanding this glaring insecurity,—notwithstanding the obvious insufficiency of the quarantine regulations, and notwithstanding the asserted malignity of the pestilential virus, which will (for it convulses, and then kills a hog in an hour,) cause birds that fly over an infected bale of cotton to drop down dead, and a priest that touches an infected altar-cloth to give up the ghost, with buboes in his armpits and groins, in less than three hours,—not one single expurgator, and not one single individual, either in Ireland, Scotland, or England, has been stricken with plague since the year 1665!

We learn from the late Dr. Harrison, that couriers are never subjected to the necessity of performing quarantine. The same gentleman told me, that the governor of the Ionian Islands was also a privileged person, and was presumed to be incapable of communicating infection! Mr. Legh also tells us, that in England the quarantine regulations are not only ineffectual, but absurd. One of the officers of the Board of Health hands up a Bible for the captain of the ship to kiss, on making oath, which, on being restored, would be sure to communicate infection, if any existed. Another produces a number of queries, to which the captain must give written answers. On this Mr. Legh remonstrated, telling the officer that nothing was so infectious as paper; but he contented himself with replying, that the orders of the Privy Council were peremptory, and must be obeyed. It would seem, therefore, that, if we have hitherto been fortunate enough to escape this dreadful calamity, it is in spite of the perilous precautions of the Privy Council.

But this paper has already extended beyond its legitimate length. I shall reserve until the next Number the few additional observations which I have to make. The exceptions which I

have hitherto taken have been physical, and derived from observations of the phenomena which the disease exhibits. The objection which I shall next take, is a moral one. I shall prove the falsehood of the doctrine of contagion, by demonstrating it to be productive of misery and crime.

